# Post-illumination cellular effects of photodynamic treatment

**DOI:** 10.1371/journal.pone.0188535

**Published:** 2017-12-04

**Authors:** Malak Charara, Artak Tovmasyan, Ines Batinic-Haberle, James Craik, Ludmil Benov

**Affiliations:** 1 Department of Biochemistry, Faculty of Medicine, Kuwait University, Kuwait City, Kuwait; 2 Department of Radiation Oncology, Duke University Medical Center, Durham, North Carolina, United States of America; Massachusetts General Hospital, UNITED STATES

## Abstract

Increased interest in clinical application of photodynamic therapy (PDT) in various medical fields poses a demand for better understanding of processes triggered by photo-treatment. Most of the work on PDT performed so far has focused on the immediate effects of photo-treatment. It is generally accepted that cellular damage occurs during light exposure and within a short period thereafter. If cells are not killed during the PDT, they might recover, depending on the extent of the photo-induced damage. Little is known, however, about the relationship between the properties of photosensitizers (PSs) and the delayed consequences of PDT. The aim of this work was to investigate cellular responses to sub-lethal photodynamic treatment and how toxicogenic potency may be affected by molecular features of the PS. Results demonstrated that for cationic porphyrin-based PSs, lipophilicity is the main factor determining the fate of the cells in the 24-hour post-illumination period. PSs with amphiphilic properties initiated oxidative reactions that continued in the dark, long after light exposure, and caused suppression of metabolism and loss of cell viability with concomitant changes in electrophoretic mobility of proteins, including caspases. Apoptotic activity was not stimulated in the post-illumination period. This study demonstrated that in PDT mediated by amphiphilic cationic metalloporphyrin PSs, even when immediate photo-damage is relatively mild, destructive oxidative processes initiated during PDT continue in the absence of light to substantially impair metabolism, and that post-illumination protein modification may modify utilization of cell death pathways.

## Introduction

Clinical application of photodynamic therapy (PDT) for treatment of various cancerous and non-cancerous diseases poses a demand for new, more efficient photosensitizers (PSs) which cause fewer side effects and are more selective. PDT is based on excitation of a PS molecule with visible light. The excited PS can participate in type I reactions, generating radicals by transfer of electrons, or in type II reactions producing singlet oxygen (^1^O_2_) by direct energy transfer to molecular oxygen (reviewed in [1]). Singlet oxygen is considered to be a primary cell damaging species in PDT because energy transfer occurs at a much higher reaction rate than electron transfer [2], it reacts with a large number of biomolecules at high reaction rates, and cells do not have enzymatic protection against ^1^O_2_.

Since PDT works via short-lived reactive species which cannot diffuse far from their site of production, the targets of PDT-induced oxidation and intracellular localization of the primary oxidative damage depend on the properties of the PS. The character of the molecular targets affected by PDT and the extent of their damage, in turn determines the mode of cell death, the nature and severity of side effects, and in general, the outcome of PDT.

We have established key molecular features that decide cellular uptake and subcellular localization of PSs, and identified parameters that direct a PS to particular subcellular compartments [3, 4]. Using Zn-porphyrin-based PSs, we identified molecular targets of photo-induced oxidative damage [5–8]. Most of the work undertaken on PDT so far, has concentrated on the immediate effects of the photo-treatment. It is accepted that cellular damage occurs during the light exposure episode and within a short period after that. Lipid peroxidation is considered the main oxidative process responsible for PDT-induced cell damage. This view is supported by the observation that compounds acting as chain-breaking antioxidants (tocopherol and trolox) can prevent PDT-induced apoptosis if present during illumination [9]. The same compounds were less efficient if added up to 10 min after the period of illumination, and were inefficient if added later than 10 min after termination of light exposure, which strengthens the view that cell fate is determined by the extent of oxidative damage caused during illumination [9]. At low PS concentrations and light doses, substantial fractions of treated cell populations can survive the initial insult. The fate of such mildly injured cells will depend on whether cell survival stress response (reviewed in [10]) or cell-destruction reactions will prevail. It has been demonstrated that sub-lethal PDT leads to activation of cellular pathways that regulate metabolism, proliferation, metastasis, and survival [11]. Activation of survival pathways is considered a major cause for resistance to anti-cancer treatment. Among the factors preventing cell survival are oxidative processes that persist long after cessation of PDT [12]. The short-lived primary ROS generated during PDT initiate autocatalytic chain reactions of peroxidation of lipids and proteins which, by chain-branching, involve more and more substrate molecules. It is not known to what extent properties of the PS influence the prevalence of cell-destructive post-illumination reactions and which properties are most significant in this regard. Since the purpose of PDT, as well as other anti-cancer treatments, is complete eradication of malignant cells, such knowledge would assist in discovery of ways to suppress cell survival processes and to augment cell-destructive post-treatment reactions. The aim of this work was to investigate how cancer cells in culture respond to sub-lethal PDT treatment and how the properties of the PS influence that response. Results demonstrate that sublethal photo-treatment with an amphiphilic cationic metalloporphyrin PS, initiated processes that continued long after the period of light exposure and which led to suppression of metabolism and loss of viability. Among the reasons for such consequences was damage of cellular proteins; PS treatment affected various classes of polypeptides, including caspases, which in turn prevented the cells from executing apoptotic death.

## Materials and methods

### Cell culture

Estrogen/tamoxifen resistant cell line pII, derived from MCF-7 breast cancer cell line by siRNA mediated silencing of the estrogen receptor [13] used in this study was provided by Dr. Y. A. Luqmani, Faculty of Pharmacy, Kuwait University. The pII cell line was chosen because in addition to being drug-resistant, it exhibits about 50% faster growth rate and is more invasive than MCF-7 [13]. Monolayer cultures of pII cells were grown in RPMI 1640 medium (Gibco) supplemented with 10% fetal bovine serum (FBS), 1% L-glutamine and 1% penicillin/streptomycin as an antibacterial agent. Cultures were kept at 37°C and 5% CO_2_, and used for experiments at 70–90% confluence. At the beginning of each experiment the growth medium was discarded, and cells were washed with phosphate-buffered saline (PBS) (Gibco). Cells were then detached by trypsinization for 2–3 min. Fresh medium with a volume of 3 ml was added to inhibit the action of trypsin. Cells were then used in experiments, and the remaining cells were sub-cultured for further use.

For counting, cells were washed with PBS and then detached by trypsinization for 2–3 min. Fresh medium (with a 10x volume of the trypsin medium) was added to inhibit the protease action. The cell suspension was centrifuged at 2500 rpm for 5 min. The supernatant was discarded, and the cell pellet was resuspended in fresh medium. The cells were diluted 10x with PBS in a small eppendorf tube. After that, 20 μl of trypan blue was added to 100 μl of the diluted sample and mixed. Cell counting was performed with a Neubauer hemocytometer to differentiate between viable and nonviable (trypan stained) cells.

For experiments, 5 x10^4^ cells per well (5x10^5^ cells/ml) were seeded into a 96-well microplate, or into 25 cm^2^ flasks and were incubated overnight to adhere. To both flasks and wells, ZnPs were added to final concentrations indicated in figures, and cells were incubated in dark for 24 h. Controls, except dark controls, had no PS addition. At the end of the incubation period cells were washed with PBS and the medium was replaced with PBS containing 0.2% glucose. Illumination was performed in PBS to avoid accumulation of toxic products due to photo-induced modifications of media components, and glucose was added to prevent decrease in ATP levels. Plates or flasks were uniformly illuminated and after illumination PBS was replaced with fresh medium. Cells were then treated according to the procedures detailed for different experiments.

### Photosensitizers and illumination

**Fig 1:**
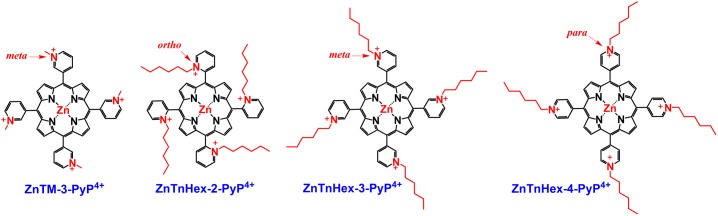
Photosensitizers used in this study. R = methyl or hexyl.

The PSs used in this study were Zn(II) *meso*-tetrakis(*N*-alkylpyridinium -2 (or -3 or -4)-yl)porphyrins, where alkyl was methyl or hexyl (Fig 1) [3]. Absorption spectra, molar extinction coefficients, lipophilicity (logP_ow_), effect of chain length and position on cellular uptake and subcellular distribution of the compounds, have been studied in detail and can be found in previous publications [3, 4, 7, 8, 14]. Additional details have been included as supporting information. All the ZnPs employed in the study are water-soluble. Concentrated stock solutions were prepared in deionized water and filter sterilized. Volumes added to cell culture were kept minimal (< 5%) and the same volume of deionized water was added to controls not containing PSs. Illumination was performed with an overhead projector (OHP-3100p, EIKI Industrial Co. Ltd.) equipped with an incandescent 300 W bulb providing fluence of 37 mW/cm^2^ at the flasks or plates. Based on preliminary investigations, an illumination time of 20 min was selected, which gave a cumulative light dose of 44.4 J/cm^2^. The temperature of the samples did not rise above 36°C during the illumination period, therefore no water filter was used.

### Treatment of cells

Except when otherwise indicated, experiments were performed in 96-well flat bottom microplates. Cells were seeded in medium and incubated overnight to adhere. The ZnPs were added to triplicate wells and cells were incubated in the dark for 24 h. At the end of incubation period the medium was replaced with PBS containing 0.2% glucose and the plates were illuminated. PBS was then replaced with fresh medium. To determine dark toxicity of the compounds, samples containing PSs but not illuminated were treated following the protocol used for the illuminated samples, except that light exposure was prevented (dark toxicity control).

Controls not containing PS but illuminated, were run in parallel with the samples. No differences were found between cells illuminated in the absence of PS and non-illuminated cells.

### MTT viability assay

The 3-(4, 5-dimethylthiazol-2-yl)-2, 5-diphenyltetrazolium bromide (MTT) assay [15] was used as a surrogate assay to determine cell viability. MTT reagent was prepared by dissolving 5 mg of MTT in 1 ml of PBS and added into each well in the dark to a final concentration of 10%. The plates were incubated for 3 h at 37°C, and 100 μl of 10% SDS in 0.01 M HCl were then added in each well. Plates were left for 24 h and absorbance was measured at 570 nm and 650 nm (as background) using a microplate reader. This assay was performed at 0 and 24 h, with zero time being immediately after illumination.

### Clonogenic assay

The effect of PDT treatment on cell survival was confirmed by a clonogenic assay [16]. The clonogenic assay provides a long-term readout of cell replicative capacity which will be a summation of all PS-mediated effects that occur in both illumination and subsequent (post-illumination) phases of a PDT protocol. Immediately after illumination, or 24 h later, cells were trypsinized, total numbers of cells counted, and 200 cells per plate were seeded. After 12 days in a CO_2_ incubator, cells were fixed and stained, and colonies were counted.

### SRB proliferation assay

Sulforhodamine B (SRB) assay was used to examine the proliferation of cells. It is based on binding of SRB to proteins of cells fixed with trichloroacetic acid (TCA) [17]. Since dead cells detach and are removed with the medium before fixation, SRB stains only viable, attached cells. SRB staining is thus considered a suitable method to estimate fully executed cell death [11].

At 0, 24, 48, and 72 hours after illumination, cells were fixed with ice cold 50% TCA to a final concentration of 10%. The plates were then incubated at 4°C for 1 h and washed five times with deionized water. Fixed cells were stained with 0.4% SRB dissolved in 1% acetic acid for 25 min. Wells were then washed five times with 1% acetic acid to remove unbound stain and air-dried at room temperature. The bound dye was solubilized with 10 mM Tris base solution with a volume equal to the volume of the original culture medium and the contents of the wells were mixed before being analyzed on a microplate reader at 510 nm, with a background measured at 690 nm.

### Cell death analysis by flow cytometry

Cell death mechanism was investigated by flow cytometry using the ANNEXIN V-FITC / 7-AAD Kit (Beckman Coulter) [18]. Cells were seeded into 25 cm^2^ sterile flasks and treated as described above. The kit was used following the manufacturer’s instructions.

### Preparation of samples for Western blotting

For each sample, cells were seeded into 25 cm^2^ sterile flasks with medium, incubated with ZnPs, and illuminated as described previously. Negative controls without PS, and dark toxicity controls, were run in parallel. In order to investigate the mechanism of cell death, some samples were treated for a 4 h period after cessation of illumination under different conditions. Some samples were kept at 37°C or on ice, while others were treated with 1% ethanolamine. Certain cell samples were kept under hypoxic conditions (< 0.3% of oxygen) in an anaerobic chamber (Plas-Labs, Inc.) for 4 h after illumination.

For homogenization, cells were detached by scraping and transferred into 15 ml tubes. Tubes were then centrifuged at 1500 rpm for 4 min, the supernatant was discarded, and the pellet was resuspended with 100 μl of homogenization buffer (50 mM NaCl, 50 mM HEPES, 5 mM EDTA, 1% Triton X-100 in distilled water to 100 ml; protease inhibitors leupeptin, aprotinin and PMSF were added immediately before use). The mixture was transferred to an eppendorf tube and kept on ice for 30 min. After that, the eppendorf tubes were centrifuged at 14,000 rpm for 20 min at 4°C. The supernatant was transferred into a new eppendorf tube for protein quantitation and the pellet was discarded. Samples were stored at -80°C for later use.

### Protein quantitation

Protein concentration was determined by Bradford’s (dye binding) protein assay using five dilutions of a standard stock solution of 2 mg/ml bovine serum albumin (BSA) prepared with distilled water for calibration. The Bio-Rad dye reagent was used in a dilution of 1:5 in distilled water. Each standard and sample was tested in duplicate. One ml of the diluted Bio-Rad reagent was taken into the cuvette and 2 μl of the tested samples were added and gently mixed. Absorbance at 595 nm was measured and protein concentrations were determined from the standard curve.

### SDS-PAGE electrophoresis

SDS-PAGE analysis was performed using standard protocols and apparatus (Mini-Protean^®^ 3 cell Bio-Rad) with 10% acrylamide separating gel and 4% acrylamide stacking gel. Samples were solubilized at 37°C in sample buffer including 5% β-mercaptoethanol. Replicate gels were run in parallel; one for protein staining (Coomassie blue stain) and the other for Western blotting. Images of gels were recorded and processed using a ChemiDoc ^™^ MP imaging system (Bio-Rad).

### Western blotting

After separation by SDS-PAGE, proteins were transferred to polyvinylidene difluoride (PVDF) membrane (Hybond-P, Amersham Biosciences) with transfer buffer (25 mM Tris and 192 mM glycine plus 10% methanol) run at 35 V overnight at 4°C.

After transfer, the blot was allowed to air-dry and the gel stained with Coomassie stain to confirm efficiency of protein transfer. The blot was wetted with methanol and distilled water and treated with 0.1% Ponceau S in 1% acetic acid, then destained with distilled water. Protein transfer uniformity was confirmed, and position of marker proteins recorded. The blot was then blocked with 5% non-fat milk in PBS and 0.05% (v/v) Tween 20 for 2h at 4°C with gentle shaking.

The blocked blot was then incubated with different primary antibodies, separately, with 5% non-fat milk in 1x PBS overnight at 4°C with gentle shaking. These antibodies were against caspase-7 (Cat no #9492; 1: 2000 dilution), cleaved caspase-9 (rabbit monoclonal Cat no #7237; 1:1000 dilution), PARP (rabbit monoclonal Cat no #9532; 1:2000 dilution) from Cell Signaling Technology, Danvers, USA, and actin (murine monoclonal anti-actin Clone AC-40; Sigma A4700 at 1:500 dilution) which recognizes a C-terminal epitope conserved in all actin isoforms and in a wide range of species, including humans (Sigma, USA).

Membranes were washed extensively with 0.05% Tween 20 in chilled PBS then incubated (2 h) with a secondary antibody peroxidase conjugated goat anti-rabbit IgG (Jackson Immunoresearch Inc., USA; at a dilution of 1:2000) for the primary antibodies against cleaved caspase-7, cleaved caspase-9 and PARP. Actin was detected using peroxidase conjugated rabbit anti-mouse IgG (Jackson Immunoresearch Inc.; with a dilution of 1:2000). Blots were then washed with PBS and 0.05% Tween 20 washing solution as described previously.

#### Detection

A chemiluminscent substrate solution (ECL, Amersham) was used for antigen imaging following the manufacturers recommendations. Images were recorded using a ChemiDoc ^™^ MP imaging system (Bio-Rad).

#### Stripping and re-probing membranes

When needed, the membranes were stripped to remove the primary and secondary antibodies to allow further probing with different antibodies of interest. Membranes were rinsed with PBS then submerged into a stripping buffer (1% SDS, 62 mM Tris pH 6.8 and 0.7% (v/v) β-mercaptoethanol). This buffer was heated in a 50°C water bath and kept with the membrane at 50°C for 20 min. The blots were washed twice with PBS for 10 min, blocked with a blocking solution for 1 h, and then re-probed with primary and secondary antibodies as described previously.

### Statistical analysis

Each experiment was repeated at least two times with three replicates. One Way Analysis of Variance (ANOVA) was performed using SigmaPlot version 11.0 and p value ≤ 0.05 was accepted as statistically significant.

## Results

**Fig 2:**
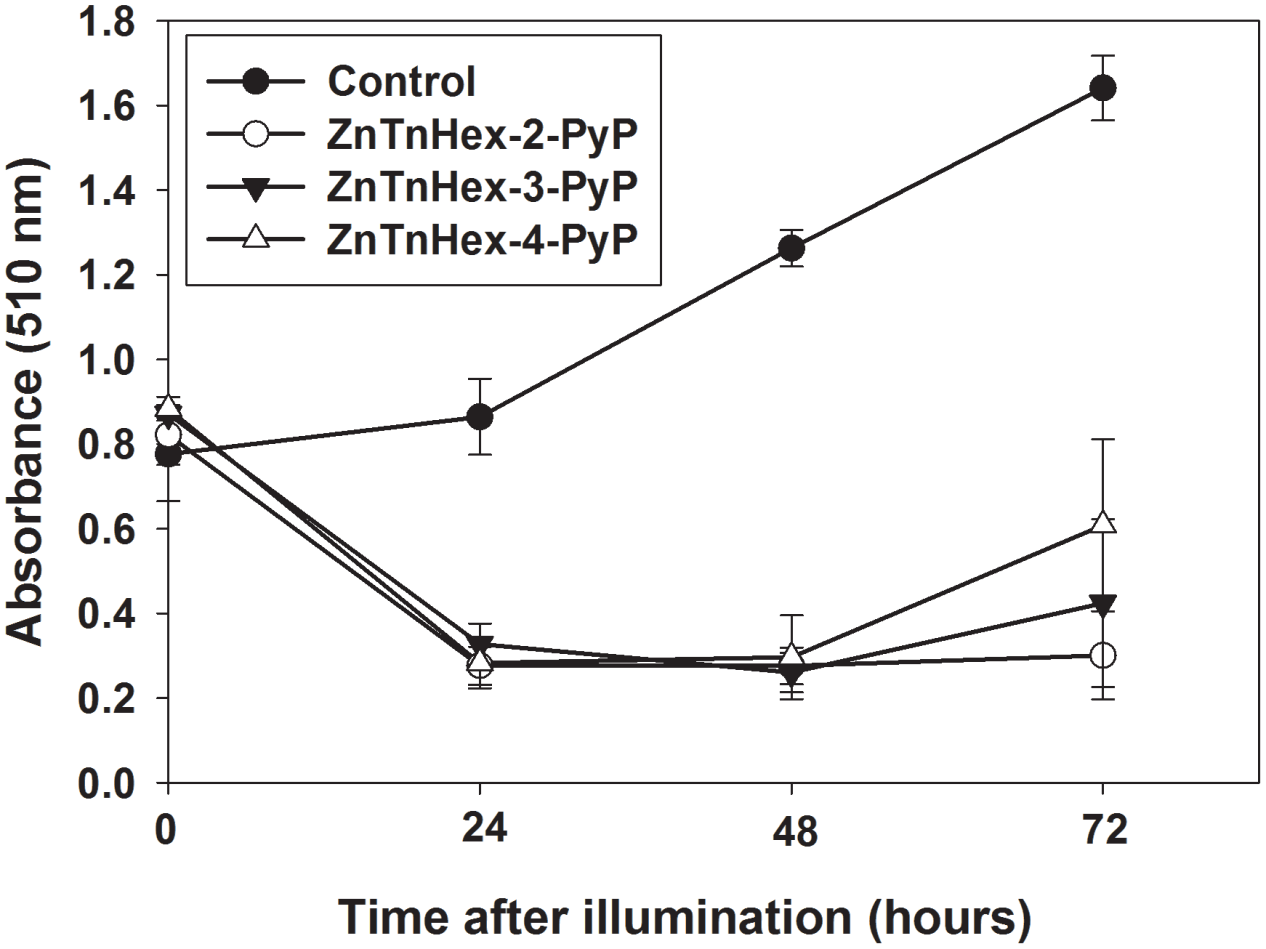
Effect of photo-treatment with ZnTnHex-PyP on cell proliferation. Cells were pre-incubated with 0.5 μM PSs for 24 h and, after replacing the medium with PBS, were illuminated for 20 min with fluence of 37 mW/cm^2^. Cell proliferation was followed by the SRB assay and measured as absorbance at 510 nm. Data is presented as mean ± SD of two separate experiments with 3 replicates each.

Our previous investigations revealed that the amphiphilic ZnP hexyl isomers are more efficient PS than their hydrophilic analogs [3]. When the effect of PDT on cell proliferation was studied, it was noticed that viable cell number dropped dramatically 24 h after illumination and then remained almost constant for the next 24 hours (Fig 2). Since cells were transferred into fresh medium without PS immediately after illumination and were kept in the dark, it was considered likely that destructive processes initiated during illumination continued after the end of the treatment. To test this assumption, cancer cell cultures were preincubated for 24 hours with varying concentrations of ZnP hexyl isomers and were then illuminated. It is important to note that the medium containing PSs was removed after preincubation, so subsequent effects were due only to compounds taken up by the cells.

No effect was observed with samples treated with ZnPs at the same concentrations but which were not illuminated (S1 Fig).

**Fig 3:**
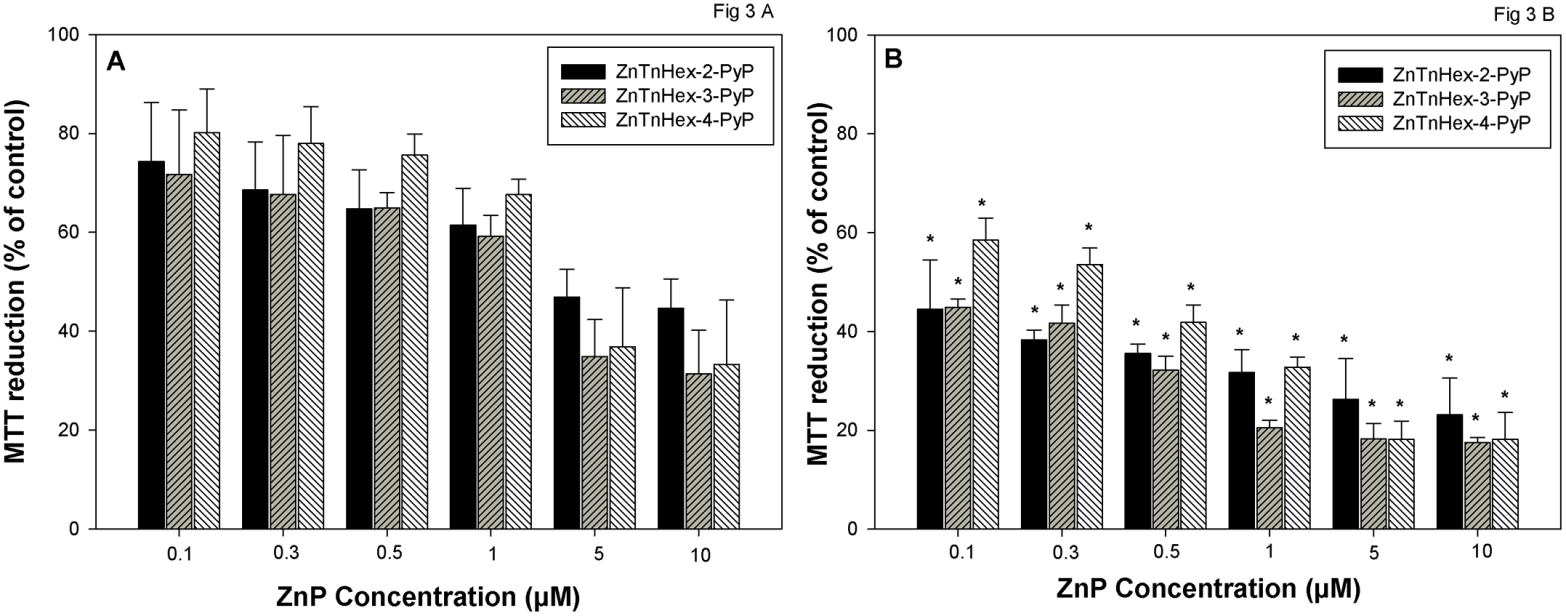
Effect of time after illumination on suppression of MTT reduction by PDT. Cells were preincubated for 24 h with ZnPs (0.1–10 μM). The medium containing ZnPs was replaced with PBS supplemented with 0.2% glucose and cells were illuminated for 20 minutes at a fluence of 37 mW/cm^2^. The MTT test was performed either immediately (**A**) or 24 hours after illumination (**B**). Data is presented as mean ± SD of two separate experiments with 3 replicates each. * Indicates statistically significant difference compared to zero hours after illumination (p < 0.05).

The surrogate viability MTT assay was used to assess the extent of cell damage immediately after illumination and 24 hours later. Fig 3 shows that for all tested ZnPs concentrations, suppression of MTT reduction increased during the 24-hour incubation period. This effect was particularly noticeable at lower concentrations of the PSs (0.1–1.0 μM), because the initial cell damage was minimal. It appeared that even if the ostensible fraction of cells impaired during the illumination did not exceed 30–40%, suppression of MTT reduction reached close to 100% after 24 hours in the dark. Similar effects were observed when cell viability was determined by the clonogenic assay (S2 Fig).

These results are in line with a previous report demonstrating that at LD_50_, a lipophilic phthalocyanine PS localizing to mitochondrial membranes, caused a time-dependent loss of metabolic activity and viability within a 24 period after illumination [11]. The authors concluded that reactions leading to suppression of metabolism and activation of cell death pathways progressed for at least 24 h after PDT [11].

Dark toxicity was assayed under identical experimental conditions, at a concentration range of ZnPs 0.1–10.0 μM, with no exposure to light (S3 Fig). Results show less than 20% suppression of MTT reduction, at the highest ZnPs concentration (10.0 μM), and ~ 10% suppression of MTT reduction by the *ortho* and *meta* ZnTnHexPyP at 5.0 μM. No dark toxicity was observed at lower concentrations of ZnPs.

Results also show small differences in photoefficiency among the three isomers, which can be attributed to differences in their physico-chemical properties and three-dimensional shapes [3]. The *ortho* isomer displayed a slightly higher capacity in generating singlet oxygen than the *meta* and *para* isomers (S4 Fig). Since the *meta* isomer, ZnTnHex-3-PyP, when applied at low concentrations, displayed intermediate photo-efficiency compared to the other two analogs, it was selected for further experiments.

**Fig 4:**
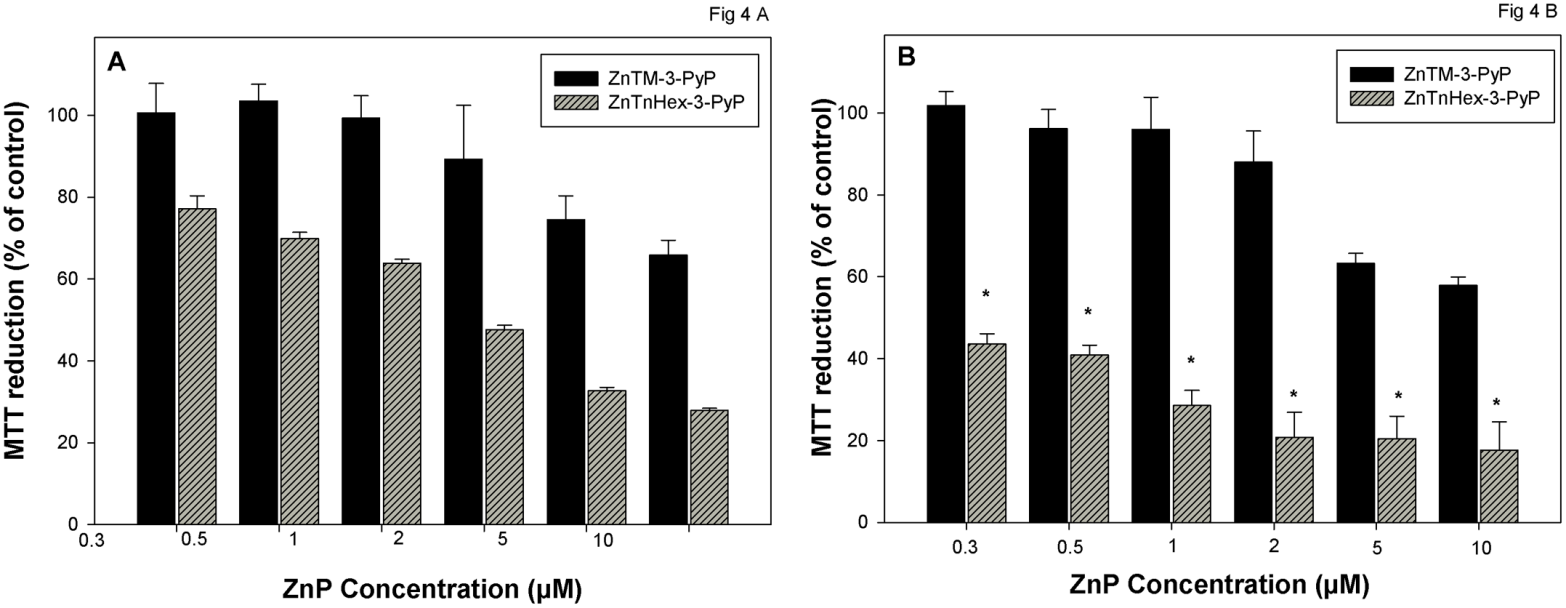
Effect of lipophilicity on the delayed cytotoxicity. Cells were preincubated with ZnTM-3-PyP or ZnTnHex-3-PyP for 24 hours before illumination. Metabolic activity of the cell population was determined with the MTT test immediately (**A**) or 24 hours after the illumination (**B**). Data is presented as mean ± SD of two separate experiments with 3 replicates each. *Indicates statistically significant difference compared to zero hours after illumination (p < 0.05).

The fact that delayed cell damage was observed at low concentrations of the PSs suggests that even a small number of ZnP molecules, if localized at specific sensitive targets, can initiate processes when illuminated which continued after the end of the photo-treatment and augmented the damage. Since cellular uptake and localization of the ZnPs depend on the structure of the PS molecule, it can be expected that the presence and significance of delayed damage will also depend on ZnP properties. Results depicted in Fig 4 show that in contrast to the amphiphilic hexyl derivative, the more hydrophilic methyl analog did not cause delayed cell damage even when applied at the highest tested concentration, 10 μM. The two cationic PSs differ by about five orders of magnitude with respect of lipophilicity [14], which dramatically affects their uptake and subcellular distribution [3]. Our previous investigations demonstrated that hydrophilic ZnPs accumulate mainly in the cytosol and the amphiphilic tetrahexyl derivatives distribute to plasma membrane and mitochondria [3, 4]. Subcellular distribution of ZnTnHex-3-PyP in endoplasmic reticulum and mitochondria of pII cells is presented in S5 Fig. This shows that the amphiphilic ZnP accumulates more in mitochondria than in endoplasmic reticulum. The weaker fluorescence of cells incubated with the hydrophilic ZnTM-3-PyP reflects its lower cellular uptake [3].

The sub-cellular distribution of ZnTnHex-3-PyP could cause photo-treatment to primarily damage lipid components of the membranes by initiating free radical chain reactions of lipid peroxidation [6]. While PDT-induced lipid peroxidation is relatively well studied [19–23], less attention has been paid to a major class of biomolecules, proteins, whose direct damage by photo-generated reactive species, or indirect damage by reactive products of lipid peroxidation, have profound biological consequences [24].

**Fig 5:**
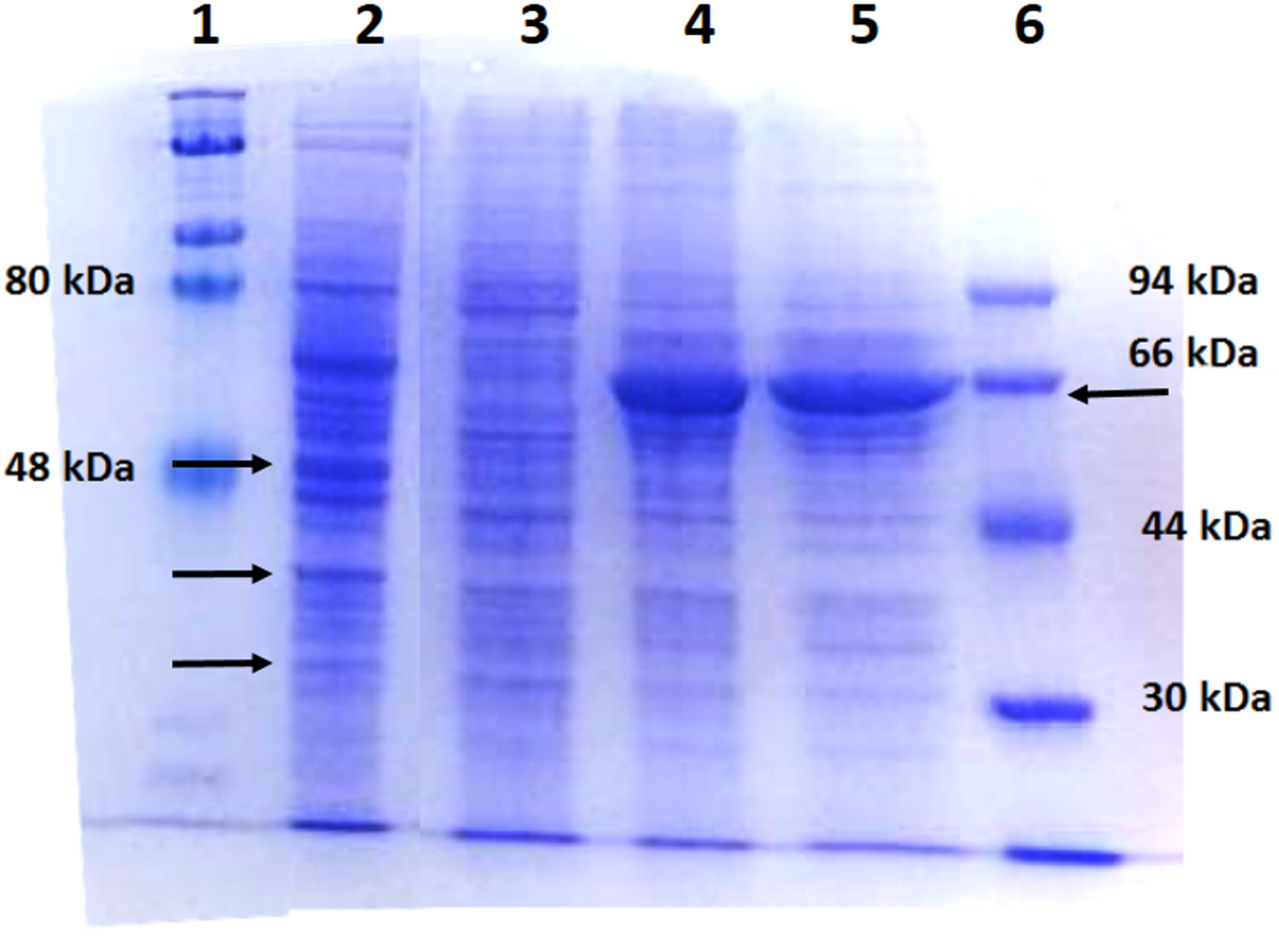
Electropherogram of proteins from cells subjected to PDT treatment with ZnTnHex-3-PyP. Cells were treated with 2 μM of ZnTnHex-3-PyP and illuminated for 20 min. The cells were disrupted either immediately after illumination or 4 and 24 hours later and then subjected to electrophoretic analysis. Ten μg of protein were applied to each lane. **Lanes:** 1) High molecular mass markers; 2) Control cells, illuminated in the absence of ZnP; 3) Cells homogenized immediately after illumination; 4) Cells homogenized 4 h after illumination and 5) Cells homogenized 24 h after illumination, 6) Low molecular mass markers. Arrows mark the positions of major protein bands that changed after PDT. A representative electropherogram is displayed; replicate experiments gave similar results.

Due to their abundance and high rate constants for reaction with singlet oxygen [25–27], proteins are regarded as primary targets for photodynamic damage [8, 28]. In addition to loss of function [5, 29], PDT-induced modifications can lead to formation of high-molecular-weight protein aggregates [2, 8, 30, 31]. In experimental systems using solutions of pure proteins, it was found that cross-linking occurs during the illumination period [8]. It is not known if similar effects take place in cells illuminated in the presence of ZnPs and if protein damage also shows time-dependence. To answer that question, cell cultures were illuminated in the presence of ZnTnHex-3-PyP, then cells were disrupted and proteins subjected to SDS-PAGE, either immediately after termination of illumination or at 4 and 24 hours later. A comparison of the electrophoretic profile at different time intervals showed that some protein bands were lost during the illumination. This loss was slightly increased with dark incubation of cells for 4 hours after illumination, but a dramatic change was the appearance of a new strong band with mobility consistent with a mass of ~ 66 kDa (Fig 5, lane 4). Keeping the cells for 24 hours after illumination had little effect on the appearance of the new ‘66 kDa’ band, but led to increased loss of bands corresponding to larger proteins (Fig 5, lane 5).

**Fig 6:**
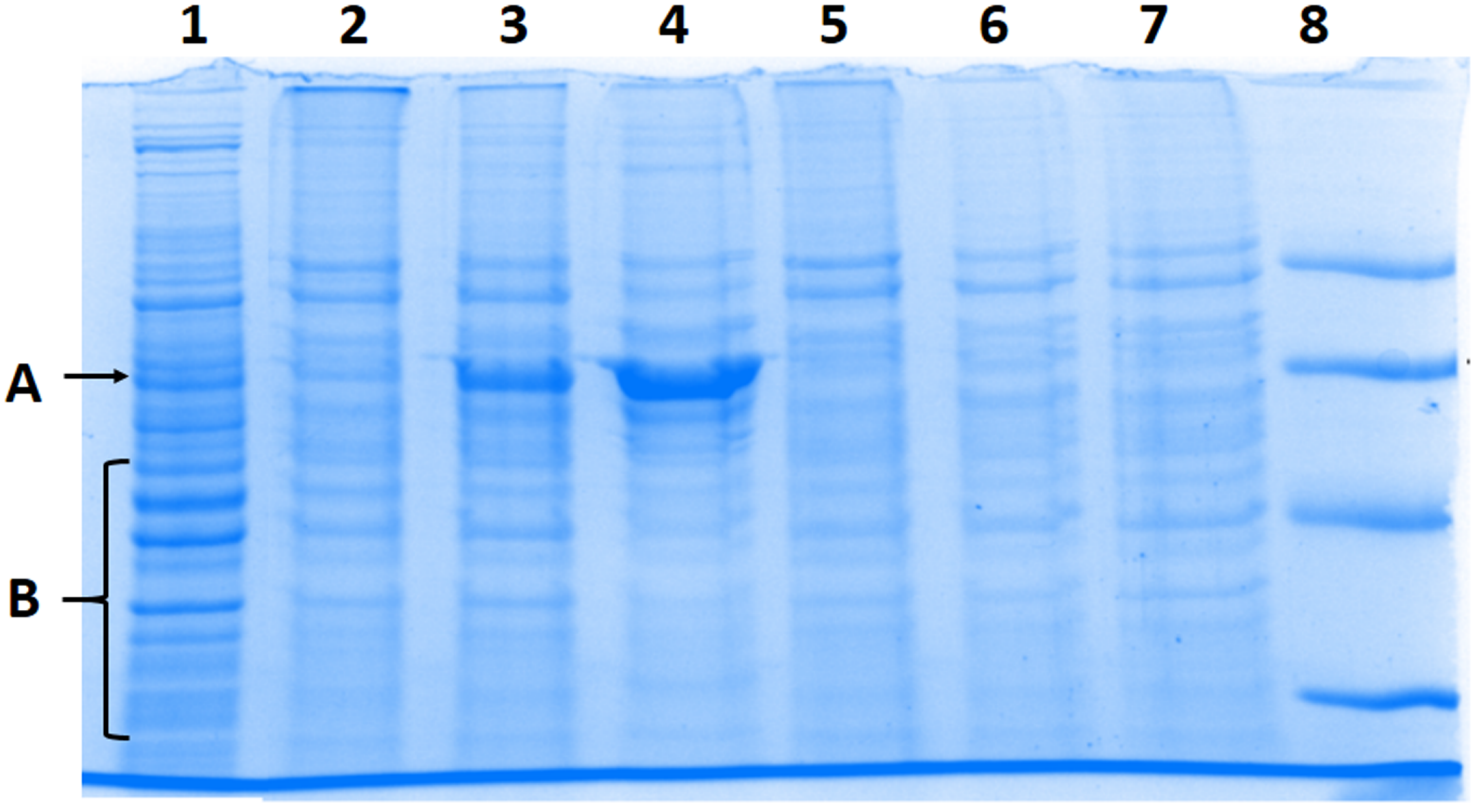
Effect of temperature, hypoxia, and ethanolamine on the electrophoretic pattern of cellular proteins. All conditions were as in Fig 5. **Lanes:** 1) Control cells illuminated in the absence of ZnP; 2) Cells homogenized immediately after illumination; 3) Cells homogenized 4 h after illumination; 4) Cells homogenized 24 h after illumination; 5) Cells kept on ice 4 h after illumination and then homogenized; 6) Cells treated with 1% ethanolamine for 4 h after illumination; 7) Cells kept under hypoxic conditions for 4 h after illumination and then homogenized; 8) Low molecular mass markers. A—position where new bands appeared; B—bands that disappeared after the illumination of cells. A representative electropherogram displayed. Replicate experiments gave similar results.

To investigate the nature of processes leading to appearance of new protein bands and disappearance of others, the cells were kept under different conditions after the illumination period. Results are presented in Fig 6. Both the appearance of new bands, and disappearance of bands corresponding to high molecular mass proteins, are prevented if cells were kept at 0°C or under hypoxic conditions after illumination (lanes 5 and 7). Similar effects of diminished post-illumination changes in protein profiles were observed if incubation after illumination was carried out in the presence of ethanolamine.

**Fig 7:**
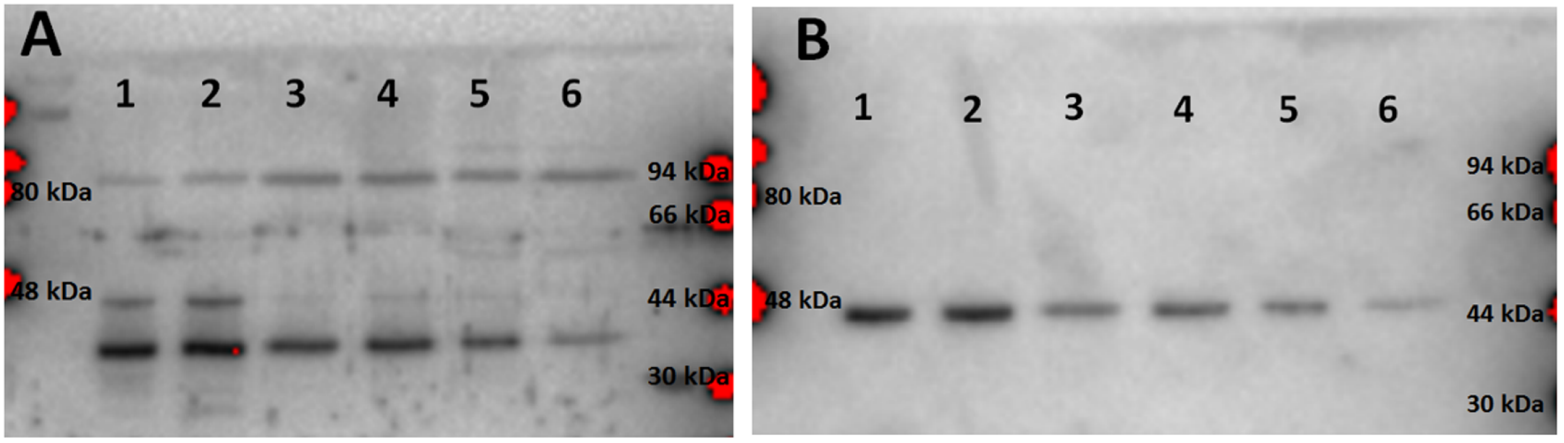
Immunoblots probed with antibodies against caspase 7 (A) and actin (B). Lanes: 1) Dark control; 2) Control cells illuminated in the absence of ZnP; 3) and 4) Cells homogenized immediately after illumination; 5) Cells homogenized 4 h after illumination; 6) Cells homogenized 24 h after illumination. Ten μg of protein were applied to each lane. A representative Western blot displayed. Replicate experiment gave similar results.

SDS-PAGE profiles indicated appearance of new SDS-PAGE protein bands after photo-treatment, with simultaneous decreases in intensity of some other protein bands. Changes in minor protein bands were difficult to identify by Coomassie staining, so Western blot analysis targeting some specific proteins was undertaken. As Fig 7 shows, the signal intensity from bands corresponding to two cellular proteins, pro-caspase 7 (panel A) and actin (panel B), gradually decreased as time progressed after illumination.

**Fig 8:**
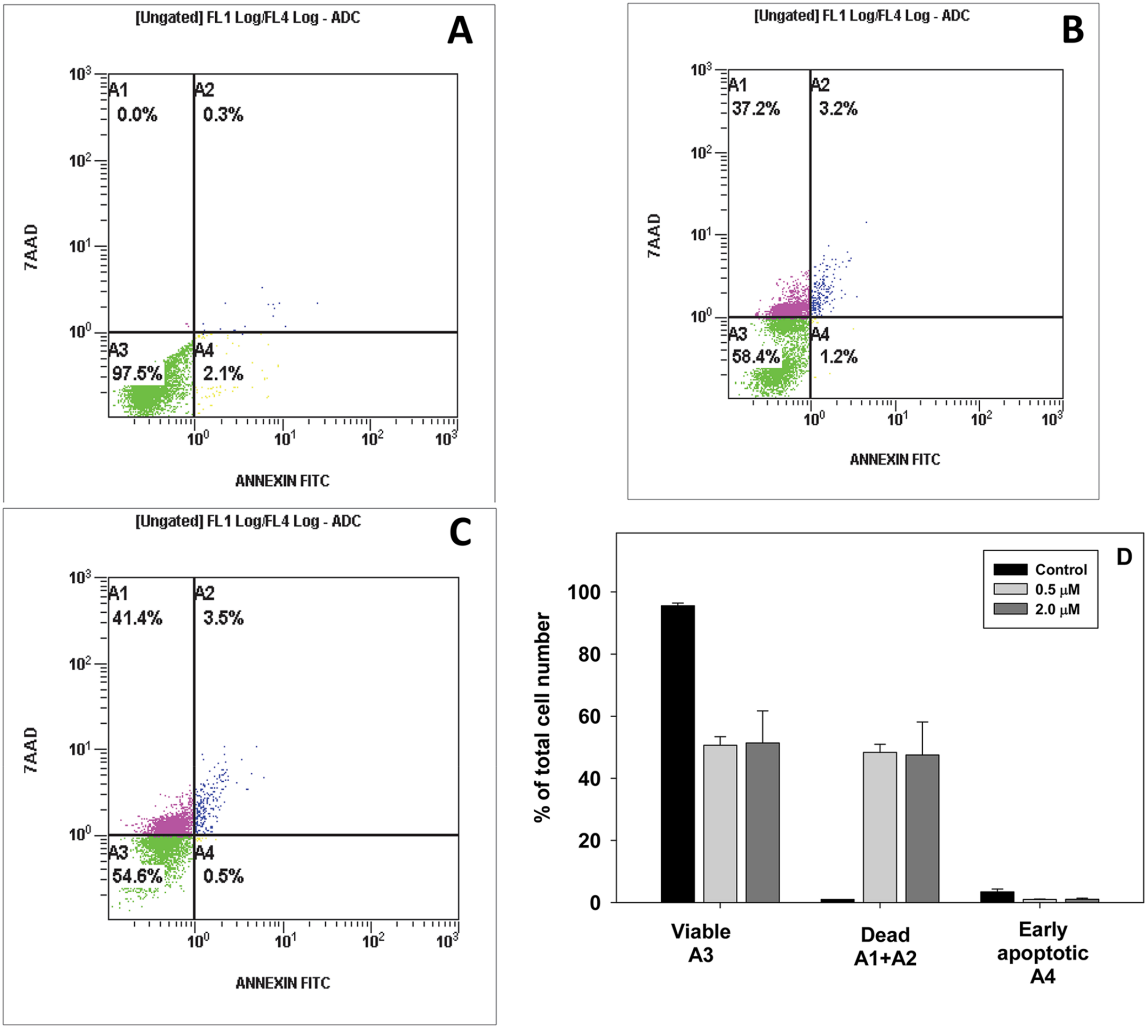
Cell death analysis. Panel A, control (untreated cells); Panel B: cells incubated with 0.5 μM of ZnTnHex-3-PyP and illuminated; Panel C, cells incubated with 2.0 μM ZnTnHex-3-PyP and illuminated. Color coding: purple (A1) and blue (A2), permeable (necrotic) cells; green (A3), viable; yellow (A4), apoptotic cells. Panel D, a bar diagram representing the distribution of cells. Mean of two separate experiments ± SD is presented.

Decrease of the band intensity of non-cleaved caspase 7 could be explained by its apoptotic proteolytic cleavage triggered by the photo-treatment [32, 33]. However, cell death analysis provided evidence that only ~ 1% of cells were apoptotic (Fig 8). Since it is known that the apoptotic process can be inhibited at higher PS doses [32], the experiment was repeated at PS concentrations of 0.5 μM (Fig 8B), and 1.0 μM, but the fraction of the apoptotic cells did not increase. The percentage of apoptotic cells did not increase when analysis was performed 4 or 24 hours after illumination.

**Fig 9:**
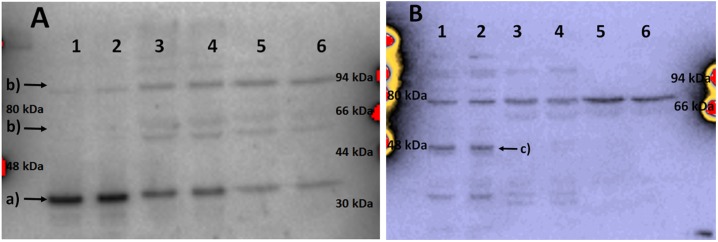
Immunoblots probed with antibodies against caspase 7 (A) and PARP (B). Arrows indicate: a) procaspase 7, b) higher molecular mass bands appearing after illumination, c) non-cleaved PARP. Lanes: 1) Dark control; 2) Control cells illuminated in the absence of ZnP; 3) and 4) Cells homogenized immediately after illumination; 5) Cells homogenized 4 h after illumination, 6) Cells homogenized 24 h after illumination. Ten μg of protein were applied to each lane. A representative Western blot is displayed. Replicate experiments gave similar results.

When cleavage of caspases and PARP [34, 35] was investigated, it was found that fading of the bands of the uncleaved proenzymes was accompanied with appearance of lower mobility bands, rather than smaller fragments expected for proteolytically processed proteins (Fig 9, caspase 7 and PARP shown, caspase 9 similar). Based on previous studies which showed appearance of high-molecular weight bands as a result of crosslinking of proteins induced by photo-activated ZnPs [7, 8], it appears very likely that crosslinking of caspases occurred when cells were illuminated in the presence of ZnTnHex-3-PyP.

## Discussion

Results of this study show that reactions, initiated by illumination of cells loaded with ZnTnHex-3-PyP, continue in the dark and cause suppression of metabolism and growth arrest. Such processes were induced by the amphiphilic hexyl, but not by its hydrophilic methyl analog, ZnTM-3-PyP. The two PSs differ in their cellular uptake and subcellular distribution. ZnTnHex-3-PyP is efficiently taken up by the cells and disperses in plasma membrane and mitochondria. Hydrophilic analogs demonstrated much lower accumulation in cells and were found mainly in lysosomes [3].

The main oxidative process triggered by PSs in biomembranes is lipid peroxidation, a self-propagating free radical chain reaction which increases the number of free radicals by chain-branching, and which generates highly reactive toxic products. Previous studies demonstrated that PDT-induced lipid peroxidation continued to increase up to 30 min after the illumination [12], and that it causes cell death [9].

In our experiments, conditions were intentionally selected to cause limited immediate photo-damage in order to explore the time-dependency of destructive processes initiated during the illumination period. Judging by the suppression of metabolic activity, toxicogenic processes causing cell damage continued after the end of the illumination. Such physiological effects were accompanied by changes in the electrophoretic pattern of cellular proteins. Low molecular weight bands were lost, and new, higher molecular weight bands appeared. This process was suppressed by lowering the temperature, by anoxia, or by adding ethanolamine. The effects of temperature and oxygen suggest that oxidative reactions initiated during the illumination continued in the dark. Since ZnTnHex-3-PyP disperses in biomembranes, membrane components are expected to be major targets of reactive species generated by the photo-excited PS. For reasons discussed elsewhere, singlet oxygen is considered to be the main cause for damage by ZnP-based PSs [1]. Membranes are particularly vulnerable to oxidative damage due to high content of unsaturated fatty acids and cholesterol, and high solubility of oxygen in lipids. It is widely accepted that lipid peroxidation products can propagate and augment oxidative damage [36]. Singlet oxygen generated by type II photodynamic reactions adds directly to double bonds, producing lipid peroxides [23, 37, 38]. In contrast, superoxide radical released by type I photodynamic reactions does not react directly with lipids, but reacts at a diffusion limited rate with [4Fe-4S]-containing proteins thus liberating “free” Fe [39]. Decomposition of lipid peroxides, catalyzed by Fe, produces radicals which trigger new lipid peroxidation reactions, thus generating more lipid peroxides [22]. This process is light-independent and, once initiated by PDT, can continue in the dark until all the substrate is oxidized [22]. Experimental evidence show that photo-generated peroxides can induce secondary chain reactions which are potentially far more damaging [22, 23]. Such chain reactions generate highly reactive electrophiles, including mono- and bifunctional aldehydes [40], which participate in addition-type reactions with the ε-amino group of lysine residues, the sulfhydryl group of cysteine residues, and the imidazole group of histidine residues [41–43]. Together with peroxidation-derived radicals, such aldehydes covalently modify and crosslink biomolecules, including proteins [44, 45]. Lysine residues are among the preferred targets for reaction with lipid peroxidation products [44]. Oxygen and temperature are limiting factors for the generation of reactive products by lipid peroxidation, which is consistent with the effects of hypoxia and low temperature on the development of post-illumination cell damage observed in this study. Protection by ethanolamine can be explained by competition with amino groups for reactions with lipid peroxidation-derived reactive products.

Cellular proteins can also be directly modified by PDT-generated reactive species [7, 8, 46]. Proteins are considered primary PDT targets due to their abundance and the high rate constants for reactions of amino acid residues with ^1^O_2_ [2, 24, 25, 30]. Using a proteomic approach, Tsaytler and coworkers identified more than 300 proteins that have been oxidatively modified by PDT [46]. Among them were metabolic enzymes and proteins involved in redox homeostasis and cell signaling.

Like lipids, proteins produce peroxides that participate in chain reactions and that can lead to peptide backbone cleavage [24]. Oxidative modification of amino acid resides, fragmentation, and crosslinking, can explain the observed changes in mobility and disappearance of bands in the PAGE. Disappearance of protein spots has been previously reported for cells subjected to PDT [47]. Using Western blotting, the same authors reported suppression of the expression of a chaperone and found protein heterodimerization. Such changes were accompanied with apoptosis of ~ 75% of the cells while only ~ 10% of the cells underwent necrosis [47].

It has been proposed that the pattern of protein oxidation reflects the intracellular localization of the PS and that protein modifications may determine the mechanism of PDT-induced cell death [28]. The same authors concluded that the oxidation of specific proteins rather than the total extent of protein oxidation determines the proclivity for initiation of a cell death pathways [28]. This might explain why a lipophilic, uncharged phthalocyanine PS induced PARP cleavage and apoptosis [46], while in our study, a cationic amphiphilic PS applied at the same concentrations, caused neither PARP cleavage nor apoptosis.

Activation of PDT-induced cell death pathways was also reported to depend on the PS dose [48]. The same PS when applied at lower concentrations or illuminated at low light fluence can induce apoptosis but higher doses, which cause extensive damage that obstruct the energy requirements for apoptosis, lead to necrosis [49, 50]. Another factor which determines the mechanism of PDT-induced cell death is the localization of the PS [50]. Under the conditions of our experiments (low ZnTnHex-3-PyP concentrations, 0.5–2.0 μM), suppression of metabolic activity immediately after the illumination was moderate. Nevertheless, cells did not show signs of apoptosis. A plausible reason for this was found in PDT-induced damage of initiator and executioner caspases. Initiator caspases exist as monomers that must dimerize for activation. Dimerization is considered crucial for the formation of the active site [51].

In contrast, effector procaspases are present as inactive dimers. They are activated by proteolytic cleavage, which after rearrangements and refolding leads to active site formation [51]. If amino acid residues critical for processing of caspase zymogens are modified by PDT, activation of procaspases would be prevented. It therefore appears that photo-induced modification of members of the apoptotic cascade can influence the mechanism of PDT-induced cell death, and consequently, PDT outcome. Thus, sensitivity towards PDT-induced modifications of initiator and executioner caspases and other members of the apoptotic cascade, and the consequential effects on accessibility of apoptosis responses, should be taken into consideration when evaluating PSs, in addition to the factors already known to prevent PDT-induced apoptosis [33, 49, 50].

## Conclusions

Destructive oxidative processes initiated during PDT continue in the absence of light. Such processes can lead to progressive impairment of metabolism and ultimately to loss of viability even when the immediate light-induced damage is relatively mild. In PDT, the mechanism of cell death depends not only on the extent of photo-damage, but also on the integrity of components of the apoptotic cascade which may be impaired during photo-treatment and further damaged by other cellular reactive species present during a subsequent post-illumination period.

## Supporting information

S1 Fig Light-independent effect of ZnPs on cell proliferation. Cells were incubated with the PSs for 24 h at a concentration of 0.5 μM. At the end of incubation period, cells were washed with PBS to remove extracellular PSs. Fresh medium was then added and cells were kept in the dark under the same conditions, and for the same time, as the illuminated samples. Cell proliferation was assessed by the SRB assay as absorbance at 510 nm. Data is represented as mean ± S.D. of two independent experiments with each sample run in duplicate.
(TIF)

S2 Fig Cell survival after illumination in the presence of ZnPs. Cells were preincubated with ZnTM-3-PyP or ZnTnHex-3-PyP for 24 hours before illumination. Cell viability was determined by the clonogenic assay. Two hundred cells were plated either immediately (A) or 24 hours after the illumination (B). Data is presented as mean ± SD of two independent experiments with 3 replicates each. As expected, due to the prolonged time necessary to form colonies, no significant difference between cells plated immediately and cells plated 24 hours after illumination was observed.
(TIF)

S3 Fig Dark toxicity of Zn-porphyrins estimated by MTT reduction. Cells were pre-incubated with ZnPs for 24 h, kept in the dark for 24 h and then assayed by the MTT test. Controls were not treated with ZnPs. Mean ± SD of two separate experiments with three replicates each is presented. Stars indicate statistically significant difference compared to control (p<0.05).
(TIF)

S4 Fig Photo-generation of singlet oxygen by *N*-alkylpyridylporphyrins. The generation of singlet oxygen was measured by the decomposition of 1, 3-diphenylisobenzofuran (DPBF) monitored as a decrease of absorbance at 415 nm. PSs (0.1 μM) were illuminated with a fluence of 3.7 mW (light intensity 10 fold lower than the intensity of illumination used in cell treatment experiments). Data is represented as mean ± S.D. of two separate experiments.
(TIF)

S5 Fig Subcellular localization of Zn-porphyrins. Confocal fluorescent images (63 x magnification) of (A) and (D), non-treated pII control cells; (B) and (E), cells incubated with ZnTM-3-PyP; (C) and (F), cells incubated with ZnTnHex-3-PyP. ZnPs were added to a final concentration of 2 μM. Incubation with ZnPs was carried out in a CO_2_ incubator at 37°C for 24 h. For confocal fluorescence microscopy the cells were seeded on sterile eight-well glass slides at 5x10^5^ cells/ml and incubated overnight to adhere. The medium was replaced with fresh medium containing ZnPs and the slides were incubated in the dark for 24 h. Controls without PS were treated the same way. After the incubation, cells were fixed with ice cold 60% ethanol. Preparations were washed twice with PBS and the chambers were removed. Slides were then mounted with Vectashield mounting medium (Vector Laboratories) and were examined using Zeiss 700 confocal fluorescence microscope. For co-localization experiments, organelle-specific stains were used (Invitrogen, Molecular Probes). Mitochondria were visualized with MitoTracker Green FM at 200 nM, the endoplasmic reticulum was stained with DIOC6 (3) iodide at 1.5 μM. The organelle tracers were first dissolved in 10% SDS to obtain 1 mM stock solutions. The tracers were then diluted to appropriate concentrations with PBS and added 30 min before fixation.
(TIF)
