# Supplementary figures and images for: Post-illumination cellular effects of photodynamic treatment
Source: PLoS One. 2017 Dec 4;12(12):e0188535. doi: 10.1371/journal.pone.0188535 (PMC5714340; doi:10.1371/journal.pone.0188535)

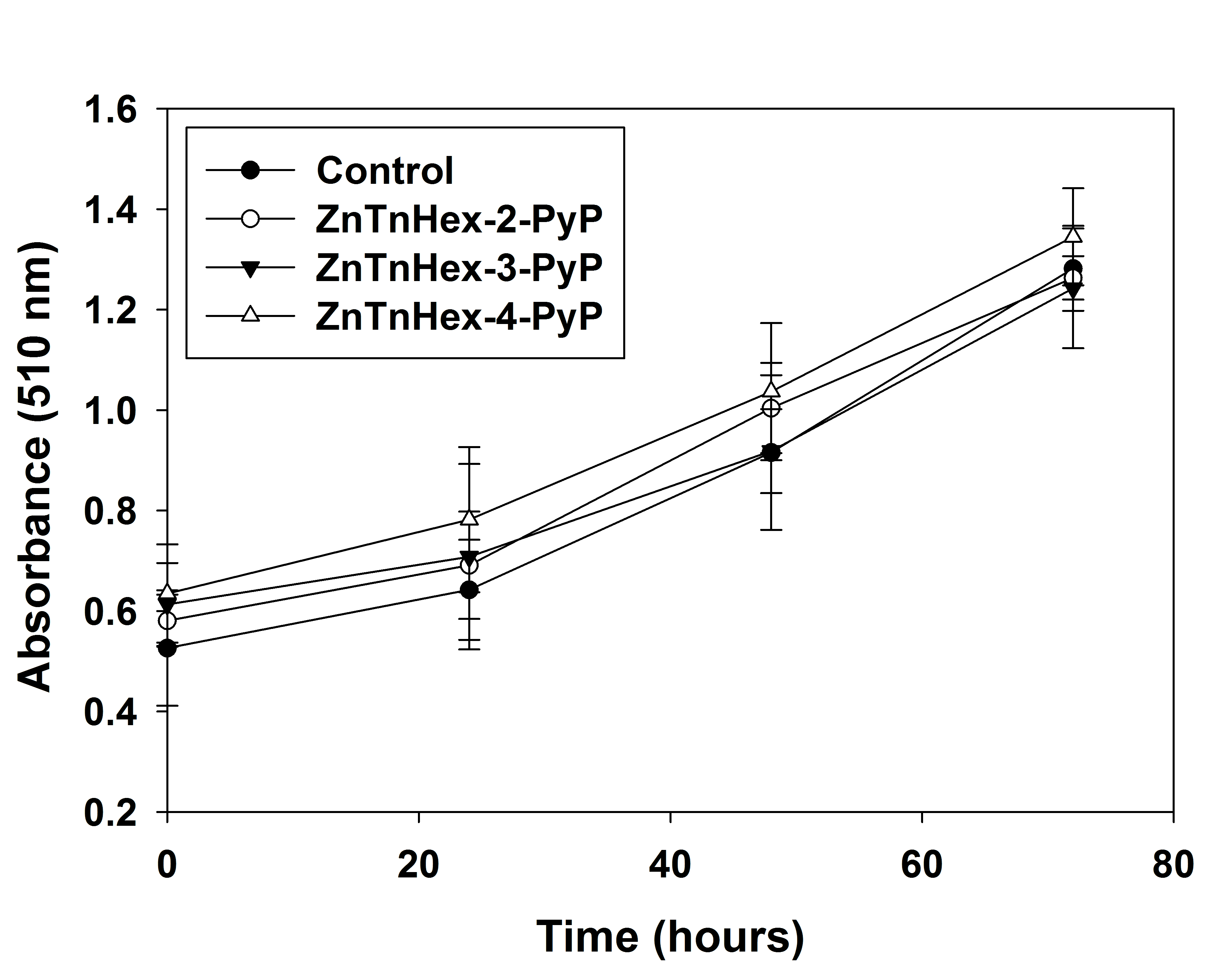

Supplement: S1 Fig — Cells were incubated with the PSs for 24 h at a concentration of 0.5 μM. At the end of incubation period, cells were washed with PBS to remove extracellular PSs. Fresh medium was then added and cells were kept in the dark under the same conditions, and for the same time, as the illuminated samples. Cell proliferation was assessed by the SRB assay as absorbance at 510 nm. Data is represented as mean ± S.D. of two independent experiments with each sample run in duplicate. (TIF) [file pone.0188535.s001.TIF]

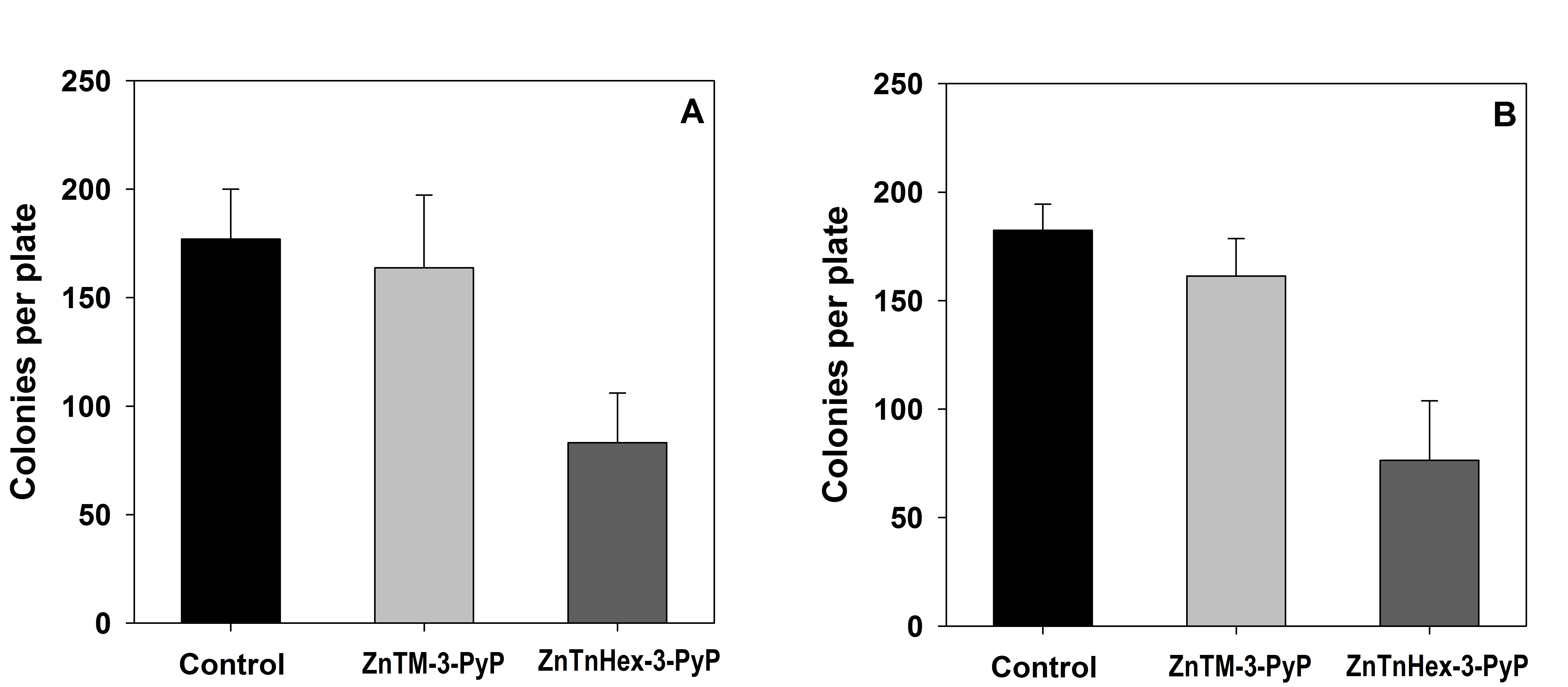

Supplement: S2 Fig — Cells were preincubated with ZnTM-3-PyP or ZnTnHex-3-PyP for 24 hours before illumination. Cell viability was determined by the clonogenic assay. Two hundred cells were plated either immediately (A) or 24 hours after the illumination (B). Data is presented as mean ± SD of two independent experiments with 3 replicates each. As expected, due to the prolonged time necessary to form colonies, no significant difference between cells plated immediately and cells plated 24 hours after illumination was observed. (TIF) [file pone.0188535.s002.TIF]

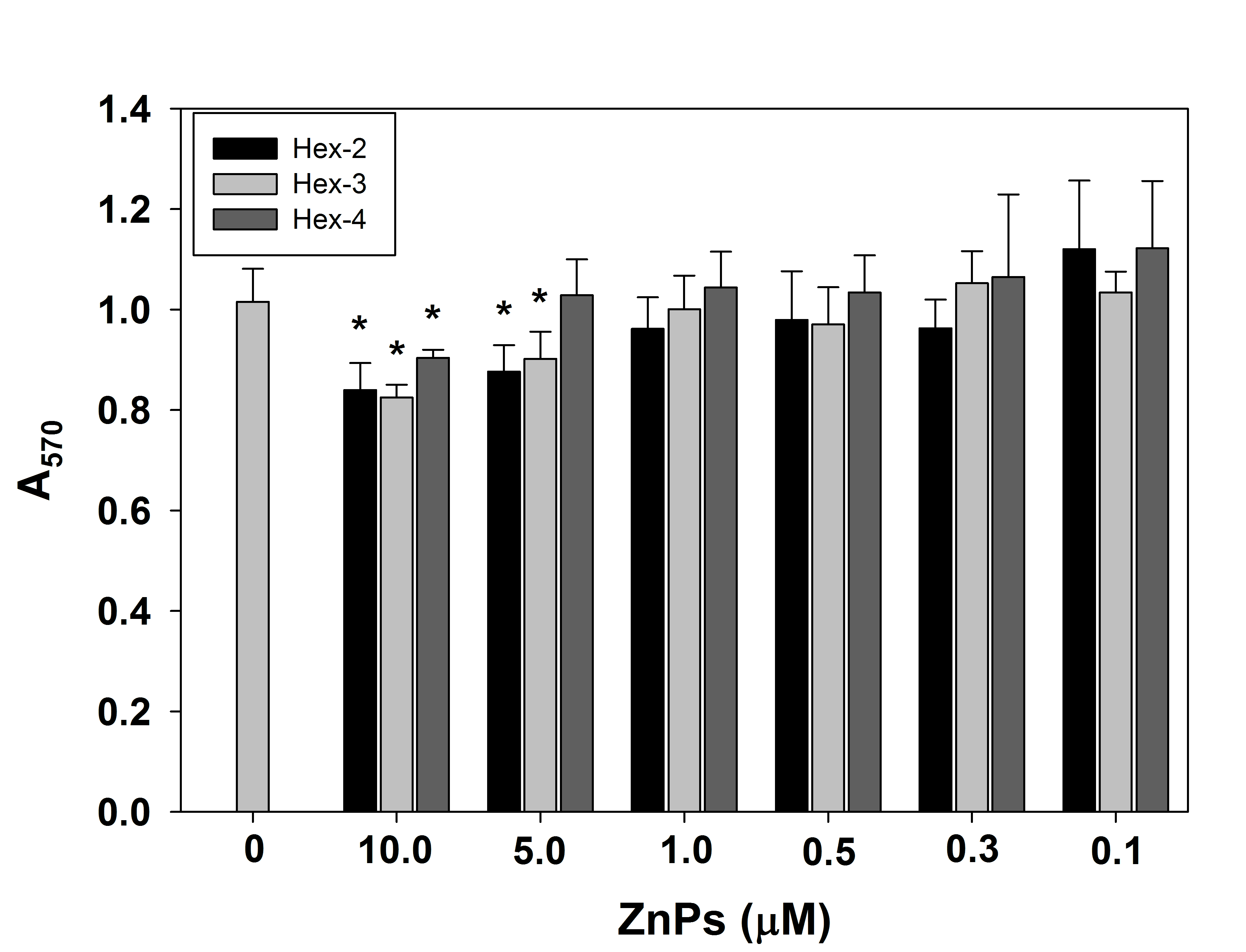

Supplement: S3 Fig — Cells were pre-incubated with ZnPs for 24 h, kept in the dark for 24 h and then assayed by the MTT test. Controls were not treated with ZnPs. Mean ± SD of two separate experiments with three replicates each is presented. Stars indicate statistically significant difference compared to control (p<0.05). (TIF) [file pone.0188535.s003.TIF]

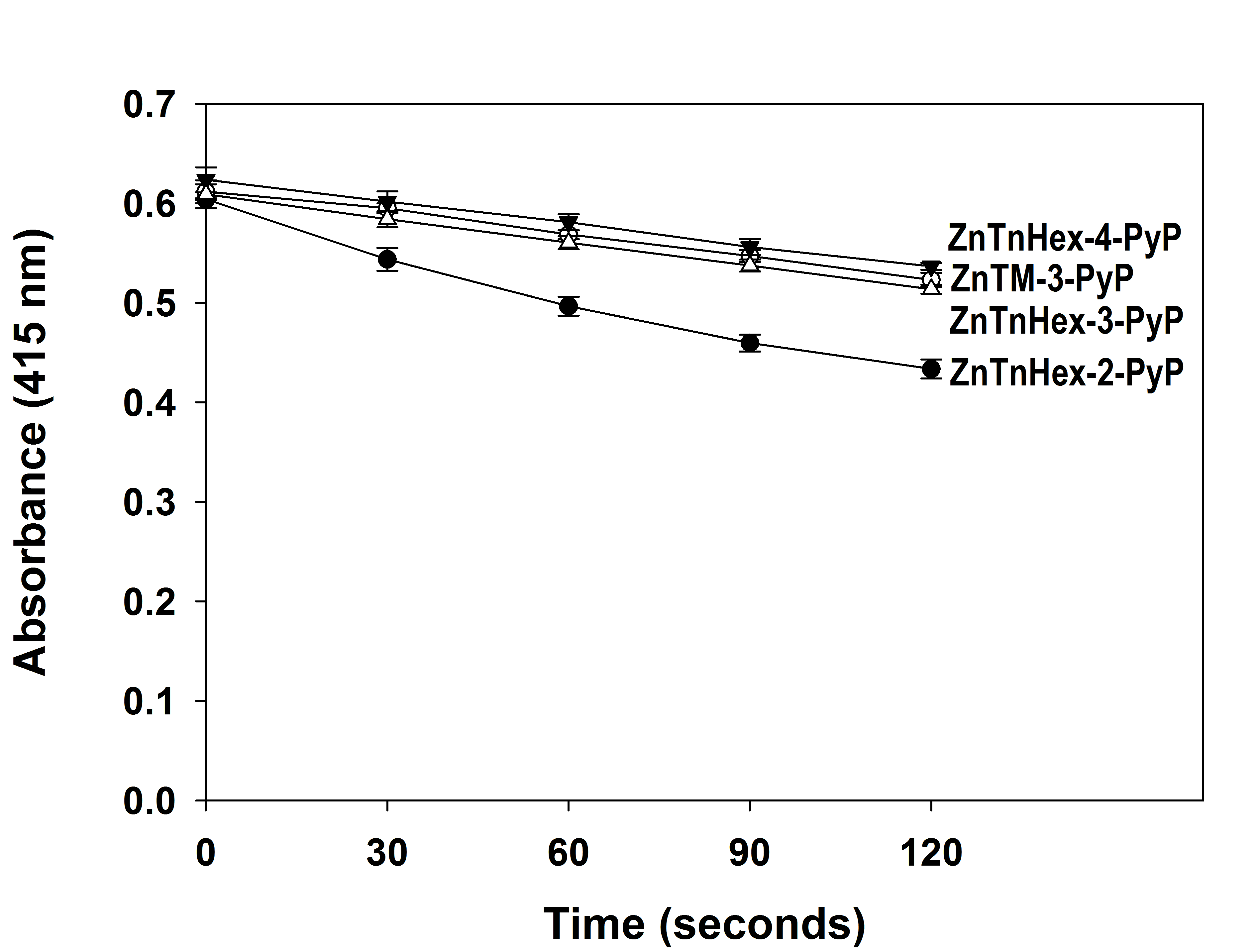

Supplement: S4 Fig — The generation of singlet oxygen was measured by the decomposition of 1, 3-diphenylisobenzofuran (DPBF) monitored as a decrease of absorbance at 415 nm. PSs (0.1 μM) were illuminated with a fluence of 3.7 mW (light intensity 10 fold lower than the intensity of illumination used in cell treatment experiments). Data is represented as mean ± S.D. of two separate experiments. (TIF) [file pone.0188535.s004.TIF]

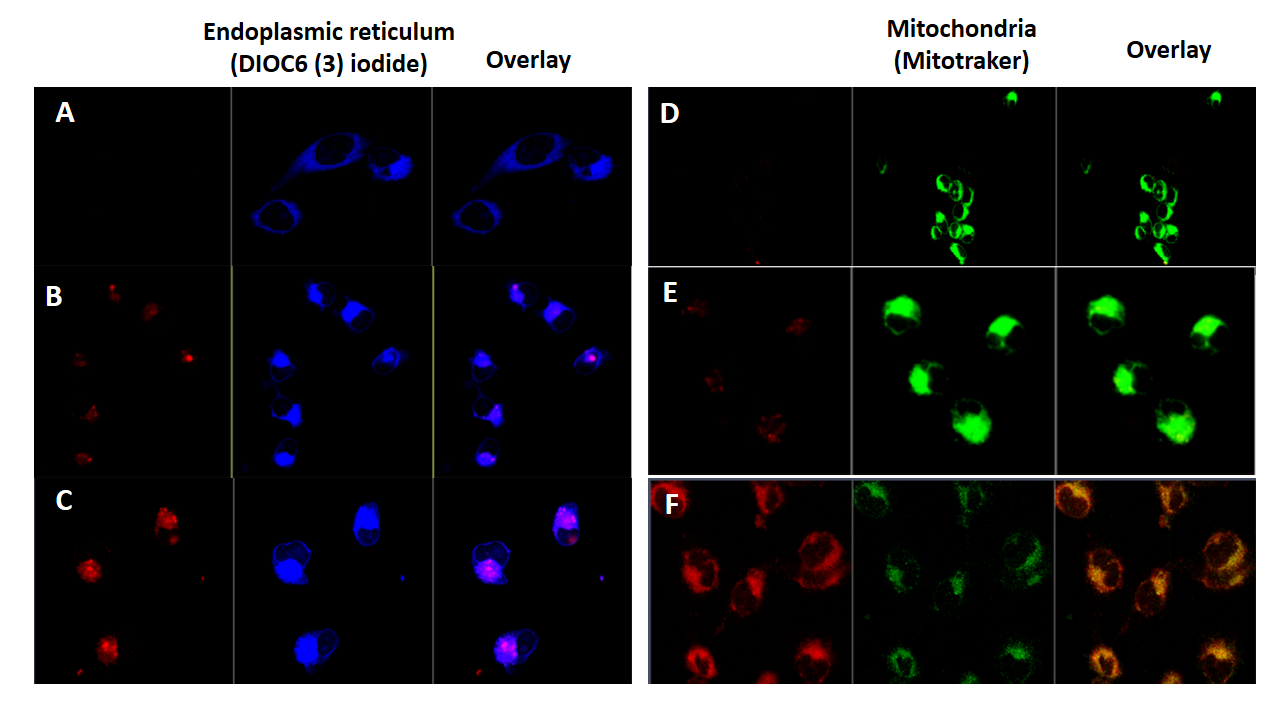

Supplement: S5 Fig — Confocal fluorescent images (63 x magnification) of (A) and (D), non-treated pII control cells; (B) and (E), cells incubated with ZnTM-3-PyP; (C) and (F), cells incubated with ZnTnHex-3-PyP. ZnPs were added to a final concentration of 2 μM. Incubation with ZnPs was carried out in a CO2 incubator at 37°C for 24 h. For confocal fluorescence microscopy the cells were seeded on sterile eight-well glass slides at 5x10^5^ cells/ml and incubated overnight to adhere. The medium was replaced with fresh medium containing ZnPs and the slides were incubated in the dark for 24 h. Controls without PS were treated the same way. After the incubation, cells were fixed with ice cold 60% ethanol. Preparations were washed twice with PBS and the chambers were removed. Slides were then mounted with Vectashield mounting medium (Vector Laboratories) and were examined using Zeiss 700 confocal fluorescence microscope. For co-localization experiments, organelle-specific stains were used (Invitrogen, Molecular Probes). Mitochondria were visualized with MitoTracker Green FM at 200 nM, the endoplasmic reticulum was stained with DIOC6 (3) iodide at 1.5 μM. The organelle tracers were first dissolved in 10% SDS to obtain 1 mM stock solutions. The tracers were then diluted to appropriate concentrations with PBS and added 30 min before fixation. (TIF) [file pone.0188535.s005.tif]
